# Supplementary material for: High metabolic load distance in professional soccer according to competitive level and playing positions
Source: PeerJ. 2022 Sep 20;10:e13318. doi: 10.7717/peerj.13318 (PMC9504445; doi:10.7717/peerj.13318)
Supplement: Supplemental Information 2 [file peerj-10-13318-s002.pdf]

ENGLISH-LANGUAGE CODEBOOK

ID: Identifying of individual match observations

PlayerPosition: playing position of soccer players. 1 = Central Backs (CB); 2 = Full Backs (FB); 3 = Center Midfields (CM); 4 = Wide Midfields (WM); 5 = Forwards (FW)

IdPlayer: Identifying of soccer players

IdTeam: Identifying of soccer teams

League: Identifying of soccer standard leagues. 1 = First Spanish Division; 2 = Second Spanish Division

PlayingTime1: Playing time of soccer players (in minutes) in the first half

PlayingTime2: Playing time of soccer players (in minutes) in the second half

PlayingTotalTime: Playing time of soccer players (in minutes) in the entire match

HMLD_m_First.half: High Metabolic Load Distance covered by soccer players (in meters) in the first half

HMLD_m_Second.half: High Metabolic Load Distance covered by soccer players (in meters) in the second half

HMLD_m_Session: High Metabolic Load Distance covered by soccer players (in meters) in the entire match

HMLD_m_First.half_min: High Metabolic Load Distance covered by soccer players per minute (in meters) in the first half

HMLD_m_Second.half_min: High Metabolic Load Distance covered by soccer players per minute (in meters) in the second half

HMLD_m_Session_min: High Metabolic Load Distance covered by soccer players per minute (in meters) in the entire match
